# Thesis advisee mistreatment and mental health: a cross-sectional study based on Structural Equation Models

**DOI:** 10.3389/fpsyg.2026.1766064

**Published:** 2026-06-10

**Authors:** Oscar Mamani-Benito, José Ventura-León

**Affiliations:** 1 Facultad de Ciencias de la Salud, Universidad Señor de Sipán, Chiclayo, Peru; 2 Facultad de Ciencias de la Salud, Universidad Privada del Norte, Lima, Peru

**Keywords:** anxiety, depression, mental health, mistreatment, stress, students, thesis

## Abstract

**Background:**

Thesis advisee mistreatment is a phenomenon that has been increasingly documented in recent years. With the emergence of validated instruments, it is now possible to measure not only its prevalence but also its association with the mental health of the university population.

**Objective:**

To analyze the relationship between thesis advisee mistreatment and depression, anxiety, and stress in university students and graduates.

**Methods:**

This was an explanatory study in which 514 undergraduate students and graduates from the three regions of Peru participated voluntarily. Data were collected using the Thesis Advisee Mistreatment Scale (EMAT) and the DASS-21 scale. Structural equation modeling was performed to analyze the relationship.

**Results:**

The latent factor mistreatment, defined by the advisor (λ = 0.91), the committee (λ = 0.96), and the administrative staff (λ = 0.83), showed significant standardized associations with stress (β = 0.59), anxiety (β = 0.59), and depression (β = 0.61). In this regard, mistreatment explained 35% of the variance in stress (R^2^ = 0.349), 35% in anxiety (R^2^ = 0.346), and 38% in depression (R^2^ = 0.377).

**Conclusion:**

Mistreatment perpetrated by all authority figures involved in the thesis process showed positive and strong associations with mental health problems. These findings suggest that a climate of persistent threat affects the confidence of university students and graduates who are working on a thesis, activating mechanisms of uncertainty and worry that, if sustained over time, could derive into symptoms of anxiety, depression, and stress. On the other hand, given the cross-sectional design of the study, causal inferences cannot be drawn, for which reason longitudinal studies are recommended in order to establish the temporal directionality of these relationships.

## 1 Introduction

The development of the undergraduate thesis takes place in a context of high academic demand and marked power asymmetry between faculty and students. Beyond constituting a requirement for obtaining the professional title or academic degree (Miyahira, 2023), this process is expected to generate an original contribution to scientific knowledge and to society (Encalada-Diaz, 2016). In this scenario, the advising relationship acquires a central role, not only in technical terms, but also in its relational and emotional dimensions.

In the international literature, the phenomenon of abusive supervision has been consistently documented due to its negative effects on the psychological well-being and performance of subordinates (Tepper, 2000; Mackey et al., 2017). In line with this, recent research suggests that the thesis development process can also be associated with students’ mental health, beyond its intellectual demands (Mavrogalou-Foti et al., 2024; Yao et al., 2025). However, empirical evidence is still limited regarding how these supervision dynamics, especially those of a dysfunctional or abusive nature, are manifested in undergraduate thesis advising.

To address this issue in greater depth, it is necessary to consider how students interact with their advisors, committee members, and administrative staff, who together form the structural and technical support of undergraduate research (Mamani-Benito, 2019, 2020). However, some of these actors have been found to have limited scientific output (Mejia et al., 2022; Mamani-Benito et al., 2019, 2020) and they apply supervision and evaluation strategies that are not very empathic, which is negatively associated with the emotional well-being of students who are working on a thesis, particularly under circumstances where experiences of mistreatment are perceived.

For instance, a study of 274 health sciences thesis students in Peru found that 13.8% said they were mistreated “often” and 6.45% said it happened “almost always” (Mamani-Benito et al., 2021a). In addition, another broader study that included business, humanities, and engineering programs found that the item with the highest score was “I have felt forced to obey all of their instructions” (Mamani-Benito et al., 2024). Likewise, when analyzing associated factors among health sciences thesis students, 8.5% reported feeling coerced by their advisor, 9.9% by the committee, and 17.6% reported lack of support from administrative staff (Mamani-Benito et al., 2021b). Lastly, evidence from a public university points to 40.2% of education thesis students reporting low levels of mistreatment, 38.3% moderate, and 21.5% high (Estrada-Araoz et al., 2023). With respect to academic supervision, asymmetries of power have been the object of international scrutiny; Grant (2005), as one example, traced how the positional power held by supervisors can silence the student voice and lock learners into a passive academic role. In a similar vein, Lee (2008) brought to light dysfunctional supervision styles that slow down student progress and feed emotional distress. All in all, these figures speak to a worrying recurrence of such episodes within university research environments.

Conceptually, thesis advisee mistreatment has been characterized as the deliberate exercise of power and the imposition of criteria, that is, verbal offense, intimidation, abuse, and belittlement, all of them as perceived by students (Mamani-Benito et al., 2021a). Such abusive conduct stems from those in charge of supervising, evaluating, and processing documentation, namely the advisor, the committee, and the administrative staff. The contact may be face-to-face or run through bureaucratic channels, and either route shapes a tense climate that slows down academic progress and corrodes psychological well-being.

No single unified theory has been put forward to cover the entire phenomenon, yet the literature gathers a handful of complementary models that account for why certain faculty members lean into attitudes and conducts tied to mistreatment. From organizational psychology, the abusive supervision framework (Tepper, 2000) holds that hostile behaviors held by a superior over time, contact of a physical nature aside, lead to emotional exhaustion, eroded self-efficacy, and psychological distress in those reporting to that superior. Within the educational sphere, the model of teacher authoritarianism, in the first place, places the professor at the center of the relationship, with the student pushed into a passive role and stripped of the right to question (Garcia and Mendoza, 2009); the authoritarian educational style, in the second place, is marked by very steep levels of demand and control, alongside low expression of affection and a thin opening for dialogue (Martin et al., 2022). The two models, anchored in rigid control and a missing bidirectional channel of communication, eat away at the confidence and the research skills of students, with downstream effects on their autonomy and creativity (Peng and Huang, 2024).

Special attention should be given to mental health within this population. The literature has placed depression, anxiety, and stress at the top of the most reported difficulties faced by university students (Leonangeli et al., 2024; Guedes et al., 2025). For the Organizacion Mundial de la Salud (2024), these three conditions sit on the list of public-health priorities, given their tie to academic failure and to risk-taking behavior. Depression has been described as a loss of interest, a deep sense of sadness, and trouble in feeling pleasure. Anxiety, on the other hand, is tied to high physiological arousal and to relentless worry. With respect to stress, it has been understood as the reaction triggered when daily demands outpace the resources available (Ooi et al., 2022; Al-Garni et al., 2025). For this reason, the present study zooms in on these three categories so as to examine how mistreatment relates to mental health.

In agreement with the tridimensional model of Lovibond and Lovibond (1995), depression, anxiety, and stress have been positioned as interlinked but conceptually distinct facets of emotional distress. Such framing lines up with the theory of negative affect, a viewpoint that admits the coexistence of a general distress factor with several specific factors (Roman et al., 2016; Sultson et al., 2024). However, the substantial overlap among these subscales has led to the proposal of bifactor models, in which a general distress factor coexists with specific components for each dimension (Peña-Tomas et al., 2025).

To date, available evidence suggests that mistreatment can seriously affect students’ emotional health during and after the thesis process. For example, in the United States, Becerra et al. (2021) explored the association between advisor characteristics and the well-being of 446 doctoral students, finding that a high-quality relationship was associated with less affective disruption under high stress. Likewise, Hunter and Devine (2016) used mixed methods with doctoral students and found that advisor experience and meeting frequency contributed to reducing emotional exhaustion.

In the United Kingdom, Mavrogalou-Foti et al. (2024) examined the predictive capacity of the student–supervisor relationship for mental health, finding that an uncertain supervision style, marked by indecision and ambiguity, was associated with higher levels of depression, anxiety, and stress. Within Belgium, Levecque et al. (2017) probed organizational factors among doctoral students and brought out that, side by side with workload and social support, the advisor’s style of supervision stood out as a leading predictor of mental health difficulties. With respect to recent Chinese evidence, perceived abusive supervision tied positively with symptoms of depression, anxiety, and psychological distress (Yao et al., 2025). The same authors traced the link to a chain mediation through the autonomy need and the professional identity of students, a finding that puts forward the weight of these psychological mechanisms.

Despite the value of the insights brought forward by such evidence, a knowledge gap remains around how mistreatment plays out during the undergraduate thesis process and among Peruvian graduates. With respect to this gap, the present work set out to estimate the link between perceived mistreatment by advisors, committee members, and administrative staff and the levels of depression, anxiety, and stress among Peruvian thesis students. On balance, the goal is to put forward solid evidence that can back the need for institutional policies, prevention programs, and psychological support strategies aimed at thesis students.

### 1.1 Hypotheses

**FIGURE 1:**
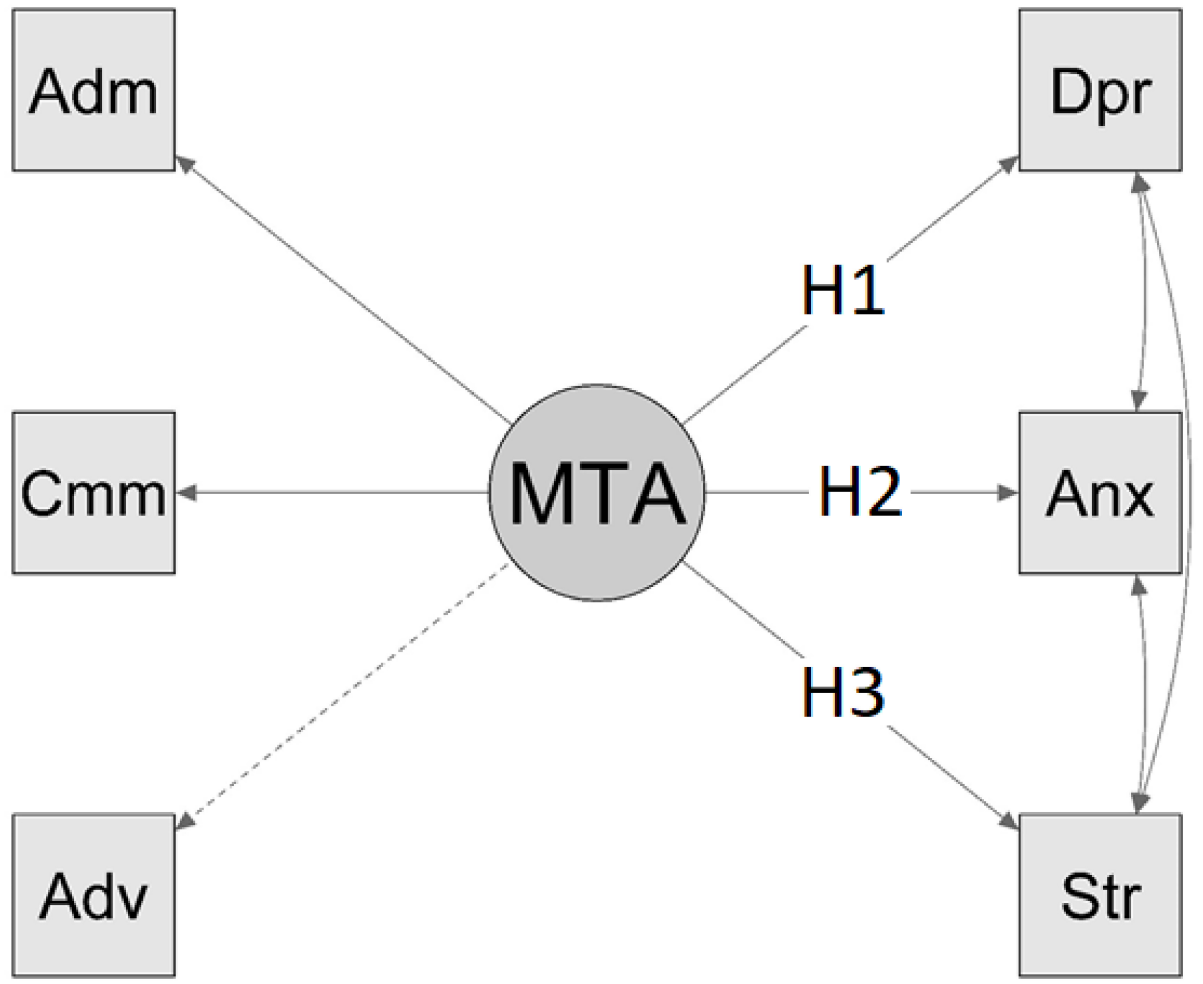
Hypothesized model.

Three hypotheses arise from the previous evidence (Figure 1 brings them together):

• H1: The latent factor Thesis Advisee Mistreatment has a direct relationship with the factor Depression.

• H2: The latent factor Thesis Advisee Mistreatment has a direct relationship with the factor Anxiety.

• H3: A direct relationship is hypothesized between the latent factor Thesis Advisee Mistreatment and the Stress factor.

### 1.2 Objective

This work set out to map the link between thesis advisee mistreatment and the dimensions of depression, anxiety, and stress in Peruvian university students and graduates, by means of Structural Equation Models (SEM).

## 2 Materials and methods

### 2.1 Design

Following Ato et al. (2013), the present design has been classified as explanatory, in that it puts under examination the link from mistreatment, the independent variable, onto depression, anxiety, and stress, the three dependent variables.

### 2.2 Participants

The sample included 514 students (269 men, 52.3%, and 245 women, 47.7%), aged between 18 and 72 years (*M* = 28.3, SD = 8.54). Within the present sample, the broad span of ages (18–72 years) speaks to the natural heterogeneity of Peruvian students and graduates, a population in which late enrollment is the rule, alongside breaks tied to work or family demands, and even the dragging out of licensure on the basis of repeated extensions of the thesis process. With respect to geographical distribution, 48.6% of participants lived in the Highlands (Sierra), 31.1% along the Coast (Costa), and the remaining 20.2% in the Jungle (Selva). The majority, namely 66.5%, were enrolled in a private university, against 33.5% who attended a public institution. By faculty, 47.9% belonged to Business Sciences (Accounting), 15.8% to other programs, 15.0% to Engineering (Civil, Systems, Industrial), 9.1% to Social Sciences (Psychology, Sociology), 7.0% to Health Sciences (Medicine, Nursing), and 5.3% to Humanities (Law, etc.). With respect to the thesis advisor, 66.0% were supervised by a male faculty member and 34.0% by a female faculty member. Their academic degrees were distributed as follows: 12.5% bachelor’s degree, 20.4% professional degree (licentiate), 40.5% master’s degree, and 26.7% doctoral degree. Finally, 51.0% of advisors were registered in RENACYT, 9.1% were not, and 39.9% of participants did not know this information.

Participants were selected through non-probabilistic intentional sampling, applying inclusion criteria such as: being a Peruvian university student enrolled or a graduate (a person who has completed all academic credits of his or her university career) who was developing a thesis to obtain the professional title; and maintaining a direct relationship with the thesis advisor for at least 4 months. Likewise, the exclusion criteria were: not granting informed consent, not having completed the form in its entirety, being a minor (<18 years), and being a graduate who had already defended the thesis or had already obtained the professional title. The latter has to do with the fact that the participant may feel grateful for having obtained the title and may tend to minimize or justify the negative behaviors of the advisor (“in the end it turned out fine”).

With respect to the recruitment procedure, this consisted of disseminating an invitation in public and private universities of the three natural regions of Peru (Highlands, Coast, and Jungle) by means of announcements on virtual platforms (mainly WhatsApp and Facebook groups), graduate networks, and direct contact with research coordinators. Each interested participant entered a unique Google Forms link that bundled, in a strict sequence, the informed consent, a sociodemographic questionnaire, and the set of assessment instruments. With a view to safeguarding the authenticity of the responses, the form was set so as to admit only one submission per email account.

### 2.3 Instrument

To assess thesis advisee mistreatment, we used a scale created by Mamani-Benito et al. (2021a) for health sciences students and later validated for students from other fields such as humanities, business, social sciences, and engineering (Mamani-Benito et al., 2024). This measure consists of 20 items distributed across three factors: mistreatment by the advisor, by the committee, and by administrative staff. Response options follow a 4-point Likert format: Never or rarely, Sometimes, Often, and Almost all the time. Regarding its psychometric properties, the cited studies report content validity based on Aiken’s V coefficient (values > 0.70 for all items), validity based on internal structure using classical and modern methods (exploratory and confirmatory factor analysis, graded response model, and measurement invariance), and very good reliability (α and ω > 0.90).

To assess anxiety, depression, and stress, we used the abbreviated DASS-21 scale developed by Lovibond and Lovibond (1995) and validated in the Peruvian context for adolescents (Contreras-Mendoza et al., 2021) and university students (Becerra-Canales et al., 2024). This instrument is composed of 21 items distributed across 3 dimensions: anxiety, depression, and stress. Response options are rated on four alternatives: 0 = did not occur to me, 1 = occurred to me a little, 2 = occurred to me quite a bit, and 3 = occurred to me very much or most of the time. In terms of psychometric properties, several studies conducted in Peru with adolescent, university, and adult populations indicate that the scale shows adequate characteristics, including content validity through Aiken’s V (V > 0.70; Contreras-Mendoza et al., 2021), structural validity through factor-analytic techniques (Becerra-Canales et al., 2024), and a very acceptable level of reliability using coefficients such as Cronbach’s alpha and Omega (>0.75; Tapullima-Mori and Chavez, 2022).

### 2.4 Procedures

Data were collected using an online survey. An electronic form was created and remained available between April 5 and May 30, 2025. This method was used for the following reasons: (a) speed and efficiency of data collection, as platforms such as WhatsApp and Facebook allow information to be gathered immediately and in real time; (b) high response rate and familiarity, since participants can respond quickly and easily from their mobile devices; (c) low cost and efficient use of resources, because data collection through social networks is more economical than traditional methods; and (d) the possibility of segmentation and targeting, as Facebook and WhatsApp make it possible to segment populations according to demographic variables, interests, or behaviors.

The first section had the informed consent, then a short sociodemographic questionnaire, and finally the study scales; participants knew their data would remain confidential and anonymous.

### 2.5 Data analysis

We computed descriptive statistics (mean, standard deviation, skewness, and kurtosis) for the mistreatment dimensions (advisor, committee, administrative staff) and the mental health dimensions (stress, anxiety, depression), and we examined their associations through bivariate correlations. With respect to the bivariate analysis, the Winsorized Pearson coefficient (r_w) was the choice, given that it performs better in the presence of outliers and non-normal distributions, two recurring features of psychological data (Ventura-León et al., 2023; Wilcox, 2011); the classical Pearson coefficient (r_p), in contrast, has been shown to inflate the strength of the correlation whenever outliers are part of the data. A Structural Equation Model (SEM) was then fitted by means of robust maximum likelihood (MLR), an estimator recommended for non-normal data (Muthén and Muthén, 2017); we assessed model fit with χ^2^, the Comparative Fit Index (CFI), the Tucker–Lewis Index (TLI), the Root Mean Square Error of Approximation (RMSEA), and the Standardized Root Mean Square Residual (SRMR) (Hu and Bentler, 1999; Kline, 2016), and we also computed coefficients of determination (R^2^) for the endogenous variables (Hair et al., 2019). Within the SEM specification, Thesis Advisee Mistreatment was set up as a latent factor underpinned by three observed parcel indicators, namely the EMAT subscale scores for advisor, committee, and administrative staff, with the loading of the first indicator fixed at 1 for identification. With respect to the three DASS-21 dimensions of depression, anxiety, and stress, these were treated as observed subscale scores rather than as latent variables; the parsimonious choice served two purposes, on one hand to keep a favorable parameter-to-sample-size ratio and on the other to align with the focal hypotheses, which targeted each DASS-21 dimension as a global construct. We freely estimated residual covariances among the three DASS-21 dimensions to account for their well-documented overlap (Lovibond and Lovibond, 1995); we performed a complete-case analysis (lavaan default), and since the online survey required a response on every item before submission, missing data was minimized. We evaluated multivariate normality by inspecting the skewness and kurtosis coefficients in Table 1; these values showed deviations from normality, which justified MLR estimation, an approach that yields standard errors and a chi-square statistic robust to non-normality.

**TABLE 1:** Descriptive statistics.

Variables	Mean	SD	Min.	Max.	g1	g2	ω
Advisor	11.75	4.71	7	28	1.27	1.2	0.91
Committee	11.56	5.05	7	28	1.13	0.57	0.94
Administration	10.78	4.83	6	24	0.9	−0.17	0.95
Stress	13.08	4.79	0	28	0.47	0.84	0.90
Anxiety	12.62	4.77	0	28	0.5	0.63	0.90
Depression	12.03	4.89	0	28	0.7	0.71	0.92

ω, omega coefficient (reliability); g1, skewness; g2, kurtosis.

### 2.6 Ethical considerations

We followed the procedures for studies with human participants in accordance with the Declaration of Helsinki, and the ethics committee of Universidad Señor de Sipán approved the study (Code: 976-CIEI).

## 3 Results

Table 1 shows the highest means for Stress (*M* = 13.08, SD = 4.79), Anxiety (*M* = 12.62, SD = 4.77), and Depression (*M* = 12.03, SD = 4.89), whereas the mistreatment dimensions ranged between *M* = 10.78 and 11.75. Skewness (g_1_ = 0.47–1.27) and kurtosis (g_2_ = −0.17 to 1.20) showed slightly right-skewed distributions and kurtosis values from flat to leptokurtic; internal consistency was adequate (ω = 0.90–0.95).

Table 2 shows that the three forms of mistreatment (advisor, committee, and administrative staff) have moderate to strong associations with the mental health variables: stress (*r* = 0.51, 0.53, 0.51), anxiety (*r* = 0.52, 0.52, 0.47), and depression (*r* = 0.53, 0.54, 0.46). Following Cohen (1988), these values (r ≥ 0.30) indicate medium to large effects, suggesting that the link between perceived mistreatment and psychological distress is substantial.

**TABLE 2:** Correlations among the variables of interest.

Variables	1	2	3	4	5
1. Advisor	–				
2. Committee	0.85	–			
3. Administration	0.69	0.78	–		
4. Stress	0.51	0.53	0.51	–	
5. Anxiety	0.52	0.52	0.47	0.84	–
6. Depression	0.53	0.54	0.46	0.80	0.83

The shaded cells indicate the variables of interest.

**FIGURE 2:**
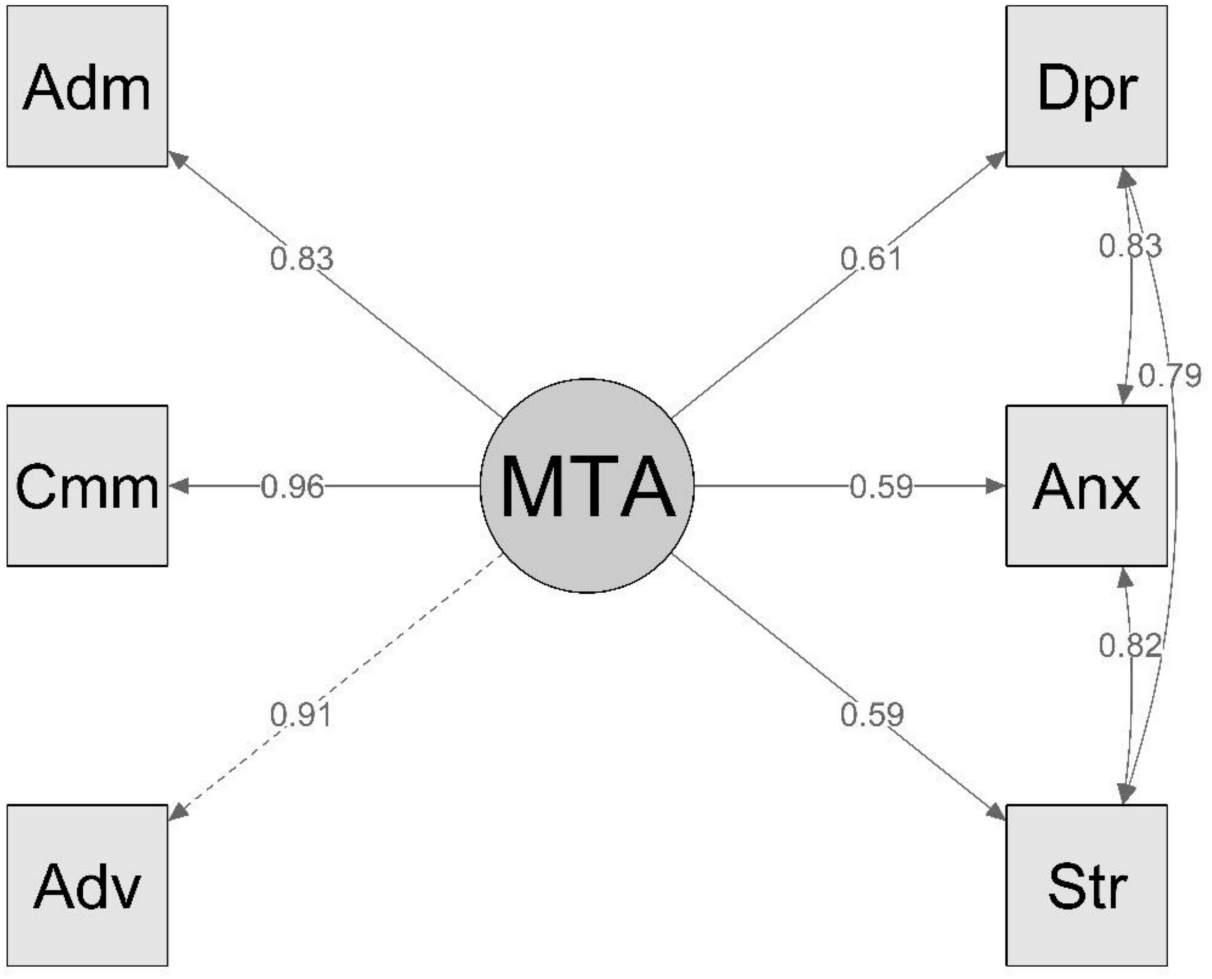
Factorial structural model. MTA, mistreatment of the thesis advisor; Adv, advisor; Cmm, committee; Adm, administration; Str, stress; Anx, anxiety; Dpr, depression.

We estimated the model with robust maximum likelihood (MLR) in a sample of 514 participants, and the latent factor mistreatment, defined by advisor (λ = 0.91), committee (λ = 0.96), and administrative staff (λ = 0.83), showed significant standardized relationships with stress (β = 0.59), anxiety (β = 0.59), and depression (β = 0.61); we also modeled residual covariances among these three outcomes (see Figure 2). Model fit was generally acceptable: χ^2^(6) = 25.21, *p* < 0.001; CFI = 0.993; TLI = 0.981; RMSEA = 0.079 (90% CI [0.052, 0.108], p_RMSEA ≤ 0.08 = 0.512); SRMR = 0.017. The mistreatment factor explained 35% of the variance in stress (R^2^ = 0.349), 35% in anxiety (R^2^ = 0.346), and 38% in depression (R^2^ = 0.377). With a value of 0.079, the RMSEA stays just under the conventional cut-off of 0.08, yet it lands on the borderline of acceptability rather than pointing to an excellent fit (Hu and Bentler, 1999). The upper bound of its 90% confidence interval (0.108) leaves room for doubt regarding close fit. Although the other indices (CFI, TLI, and SRMR) fall within recommended thresholds and support the structural relationships, we interpret them with caution.

## 4 Discussion

In the university setting, where the thesis demands not only high methodological rigor but also an original contribution to the academic community (Miyahira, 2023; Encalada-Diaz, 2016), students’ mental health emerges as a critical aspect that cannot be overlooked. In this sense, the relational and emotional dimensions play a key role during university research processes, for which reason this study aimed to analyze how mistreatment by advisors, committee members, and administrative staff is related to depression, anxiety, and stress in Peruvian students and graduates who are working on a thesis.

First, the results show that the mean levels of mistreatment reported from advisors (*M* = 11.56), committee members (*M* = 11.75), and administrative staff (*M* = 10.78) are notably similar. This finding extends what was observed by Mamani-Benito et al. (2021a), since in that study with health sciences student’s administrative mistreatment predominated, followed by committee and, lastly, advisor mistreatment. Therefore, by including a more diverse sample from different academic areas, we observed a more balanced perception of mistreatment, suggesting that authoritarian practices described as intentional manifestations of power and imposition of criteria (Mamani-Benito et al., 2021b) are widespread across various university contexts. This homogeneous distribution of mistreatment among the three actors coincides with what was described by Grant (2005) and Lee (2008), who documented that power asymmetries in academic supervision do not reside exclusively in the principal figure of supervision, but rather extend to the entire institutional ecosystem that surrounds the thesis student.

Likewise, the scores for stress (*M* = 13.08), anxiety (*M* = 12.62), and depression (*M* = 12.03) were very similar, which is consistent with the tridimensional model of Lovibond and Lovibond (1995) in showing generalized and overlapping emotional distress, as anticipated when describing depression, anxiety, and stress as independent yet correlated dimensions. This reinforces the idea that, in highly demanding academic settings, experiences of mistreatment may be associated with a general negative affect. Although constructs such as autonomy or trust were not directly measured, previous studies (Peng and Huang, 2024) suggest that these could be underlying mechanisms that future research should examine.

With regard to H1, a strong and direct relationship of mistreatment with depression was observed (β = 0.61). In line with the literature for example, Mavrogalou-Foti et al. (2024) found that conflictive supervision explained 26% of the variance in depression among doctoral students; in a consistent manner, the meta-analysis by Mackey et al. (2017) concluded that abusive supervision presents one of the most robust associations with depression among all the psychosocial predictors evaluated. More recently, Yao et al. (2025) reported in Chinese students a significant correlation between abusive supervision and depressive symptoms (β = 0.39, *p* < 0.001). These data confirm that each act of mistreatment in supervision is associated with a clinically significant risk factor. However, it is important to note that the present findings only show a strong association between mistreatment and depression, but not directly possible mechanisms such as ruminative thoughts or hopelessness. Based on previous literature (Fox et al., 2024; Vilchez-Cornejo et al., 2020), it could be assumed that the lack of empathy or rigid control techniques may increase these cognitive patterns; however, this constitutes a theoretical interpretation that requires direct evaluation in future studies.

Regarding H2, a strong and direct relationship with anxiety (β = 0.59) was also confirmed. As described earlier, anxiety involves excessive physiological arousal and persistent worry (Ooi et al., 2022; Al-Garni et al., 2025). Previous research suggests that mistreatment can generate constant uncertainty, which could trigger neurophysiological alarm responses (Tolin et al., 2023). Although the present study did not directly evaluate these physiological mechanisms, the observed association between mistreatment and anxiety is consistent with such interpretation. In line with studies in youth and adolescents (Assari and Moghani Lankarani, 2018; Makowska and Wyleżałek, 2021), these experiences of academic violence are related to greater excessive worry, insomnia, and somatic symptoms.

Finally, with regard to H3, the present data confirm a strong direct relationship between mistreatment and stress (β = 0.59), for which reason it is possible to assume that constant disqualification and the imposition of rigid criteria increase psychological pressure. In this regard, the international evidence supports this interpretation, as revealed by the study of Mackey et al. (2017), where it was found that abusive supervision is consistently associated with emotional exhaustion (a central component of chronic stress) with a moderate to large effect size. In addition, Yao et al. (2025) documented that the autonomy need frustrated by abusive supervision acts as a mediator of psychological distress. In this context, it is important to recall that, as Estrada-Araoz et al. (2023) pointed out, in educational contexts mistreatment in its various levels is correlated with hostile research climates, increasing emotional pressure and limiting participation and the expression of ideas (Syahid et al., 2023).

In this sense, the present research offers key strengths. First, it represents the first study in the Peruvian context to comprehensively incorporate the relational and emotional dimensions of mistreatment in undergraduate theses. It also includes participants from several academic areas, which facilitates the design of interventions tailored to specific contexts. The use of validated instruments and SEM analyses strengthens internal validity and supports the adoption of factorial models for the study of depression, anxiety, and stress (Peña-Tomas et al., 2025). Finally, it generates robust evidence to support the development of policies and support programs, in line with the need to promote institutional change.

Nonetheless, this study has certain limitations that should be considered. Because it uses a cross-sectional design, it is not possible to establish definitive causality; future research should therefore implement longitudinal designs to track the temporal evolution of the effects of mistreatment. Additionally, the use of online surveys may introduce self-report bias, which makes it advisable to complement them with in-depth interviews. Moderating variables that could mitigate the impact of these experiences, such as social support, resilience, and coping strategies, were not included in the present model. Finally, given that the sample is not representative of all regions of Peru, it is recommended that future studies use probabilistic sampling techniques to improve the generalizability of the findings. An additional methodological caveat is that, although the global fit indices were generally acceptable, the RMSEA value (0.079) approached the upper bound of what is conventionally regarded as acceptable, indicating that the model’s close fit cannot be firmly established. Future studies with larger and more heterogeneous samples could test alternative specifications (e.g., fully latent models or bifactor representations of the DASS-21) that may yield improved model fit. Lastly, several potentially confounding variables and explanatory mechanisms were left out of the present model, namely mental health history, academic load, socioeconomic stress, social support, resilience, thesis stage, rumination, hopelessness, neurophysiological activation, restriction of autonomy, and loss of trust. This limits the interpretation of the observed associations and keeps the underlying processes at a speculative level; for this reason, future longitudinal studies should incorporate direct measures of these variables in order to empirically contrast the proposed mechanisms.

## 5 Conclusion

The present findings indicate suggest that mistreatment by any authority figure during the thesis is related to mental health problems. The results indicate that persistent threat erodes the thesis student’s confidence and triggers uncertainty and excessive worry, which could be expressed suggest that persistent threat could be associated with a decrease in the thesis student’s confidence and with the emergence of uncertainty and excessive worry, variables that, in turn, could be expressed in symptoms of anxiety, depression, and stress. In response, universities urgently need preventive policies and psychological support programs for students and graduates who are working on their theses under adverse conditions, since addressing and preventing these practices in the university sphere is decisive in order to protect the mental health and emotional well-being of students and graduates during the university research process.

## Data Availability

The datasets presented in this study can be found in online repositories. The names of the repository/repositories and accession number(s) can be found below: https://doi.org/10.5281/zenodo.17904872.

## References

[B1] Al-Garni A. M. Shati A. A. Almonawar N. A. Alamri G. M. Alasmre L. A. Saad T. N. et al. (2025). Prevalence of depression, anxiety, and stress among students enrolled at King Khalid University: a cross-sectional study. *BMC Public Health* 25:354. 10.1186/s12889-025-21277-7 39875847 PMC11773868

[B2] Assari S. Moghani Lankarani M. (2018). Violence exposure and mental health of college students in the United States. *Behav. Sci.* 8:53. 10.3390/bs8060053 29882926 PMC6027217

[B3] Ato M. López J. Benavente A. (2013). Un sistema de clasificación de los diseños de investigación en psicología. *Anal. Psicol.* 29 1038–1059. 10.6018/analesps.29.3.178511

[B4] Becerra M. Wong E. Jenkins B. Pressman S. (2021). Does a good advisor a day keep the doctor away? How advisor-advisee relationships are associated with psychological and physical well-being among graduate students. *Int. J. Commun. Well-Being* 4 505–524. 10.1007/s42413-020-00087-2

[B5] Becerra-Canales B. Hernandez-Huaripaucar E. Cordova-Delgado M. Pastor-Ramirez N. Melgarejo-Angeles W. Balbuena-Conislla H. et al. (2024). Structural validity and reliability of the depression anxiety stress scale in peruvian university students. *Rev. Hosp. Psiquiátrico Habana* 21 1–15.

[B6] Cohen J. (1988). *Statistical Power Analysis for the Behavioral Sciences*, 2nd Edn. Hillsdale, NJ: Lawrence Erlbaum.

[B7] Contreras-Mendoza I. Olivas-Ugarte L. De la Cruz-Valdiviano C. (2021). Escalas abreviadas de Depresión, Ansiedad y Estrés (DASS-21): validez, fiabilidad y equidad en adolescentes peruanos. *Rev. Psicol. Clín. Niños y Adolesc.* 8 24–30.

[B8] Encalada-Diaz M. (2016). El valor de una tesis. *Acta Ortopedica Mexicana* 30:51. 27846349

[B9] Estrada-Araoz E. Quispe-Mamani Y. Quispe-Aquise J. Cruz-Huisa R. (2023). Percepción del Maltrato a los Asesorados de Tesis en una Universidad Pública: Un Estudio Exploratorio. *J. Law Sustain. Dev.* 11:e864. 10.55908/sdgs.v11i4.864

[B10] Fox S. W. Morgan V. R. Strait G. G. et al. (2024). Childhood maltreatment, mindfulness, and the mediating role of rumination in college students. *Curr. Psychol.* 43 35872–35888. 10.1007/s12144-024-07033-x

[B11] Garcia Y. Mendoza B. (2009). El autoritarismo y su expresión en la formación docente. *Laurus* 15 71–93.

[B12] Grant B. M. (2005). Fighting for space in supervision: fantasies, fairytales, fictions and fallacies. *Int. J. Qual. Stud. Educ.* 18 337–354. 10.1080/09518390500082483

[B13] Guedes L. Engstrom M. Schneider B. Fonseca C. Lindberg M. Schroder J. et al. (2025). Symptoms of anxiety and depression among health and social science students: A multicenter study. *Heliyon* 11:e41957. 10.1016/j.heliyon.2025.e41957 39897836 PMC11786828

[B14] Hair J. F. Black W. C. Babin B. J. Anderson R. E. (2019). *Multivariate Data Analysis*, 8th Edn. Cengage Learning.

[B15] Hu L. T. Bentler P. M. (1999). Cutoff criteria for fit indexes in covariance structure analysis: Conventional criteria versus new alternatives. *Struct. Equ. Model. Multidiscipl. J.* 6 1–55. 10.1080/10705519909540118

[B16] Hunter K. Devine K. (2016). Doctoral students’ emotional exhaustion and intentions to leave academia. *Int. J. Doctoral Stud.* 11 35–61. 10.28945/3396

[B17] Kline R. B. (2016). *Principles and practice of structural equation modeling*, 4th Edn. Guilford Press.

[B18] Lee A. (2008). How are doctoral students supervised? Concepts of doctoral research supervision. *Stud. Higher Educ.* 33 267–281. 10.1080/03075070802049202

[B19] Leonangeli S. Michelini Y. Rivarola G. (2024). Depression, anxiety and stress in college students before and during the first three months of COVID-19 lockdown. *Rev. Colombiana Psiquiatr.* 53 284–294. 10.1016/j.rcp.2022.04.008 35663410 PMC9135671

[B20] Levecque K. Anseel F. Beuckelder A. Van der Heyden J. Gisle L. (2017). Work organization and mental health problems in PhD students. *Res. Police* 46 868–879. 10.1016/j.respol.2017.02.008

[B21] Lovibond P. F. Lovibond S. H. (1995). The structure of negative emotional states: comparison of the Depression Anxiety Stress Scales (DASS) with the Beck Depression and Anxiety Inventories. *Behav. Res. Ther*. 33 335–343. 10.1016/0005-7967(94)00075-u 7726811

[B22] Mackey J. D. Frieder R. E. Brees J. R. Martinko M. J. (2017). Abusive Supervision: A Meta-Analysis and Empirical Review. *J. Manage.* 43 1940–1965. 10.1177/0149206315573997

[B23] Makowska M. Wyleżałek J. (2021). A qualitative study of the mistreatment of medical students by their lecturers in polish medical schools. *Int. J. Environ. Res. Public Health* 18:12271. 10.3390/ijerph182312271 34885997 PMC8657188

[B24] Mamani-Benito O. (2019). La asesoría de tesis en pregrado: una labor que requiere un nuevo enfoque. *Rev. Med. Herediana* 30 124–125. 10.20453/rmh.v30i2.3555

[B25] Mamani-Benito O. (2020). Limitada producción científica de la comunidad universitaria: Rol del jurado de tesis. *Rev. Med. Herediana* 31 134–135. 10.20453/rmh.v31i2.3779

[B26] Mamani-Benito O. Rojas-Zegarra M. E. Carranza Esteban R. F. Caycho-Rodríguez T. Vilca L. W. Lingán-Huamán S. K. (2024). New psychometric evidence for the thesis advisor abuse scale (EMAT) in Peruvian university students based on classic and modern procedures. *Heliyon* 10:e28475. 10.1016/j.heliyon.2024.e28475 38560100 PMC10979084

[B27] Mamani-Benito O. Tito-Betancur M. Corrales-Reyes I. Mendoza D. Rivera-Berrios L. Mejia C. (2021b). Factores asociados al maltrato hacia los tesistas de ciencias de la salud en el Perú. *Rev. Cubana Med. Militar* 50:e02101613.

[B28] Mamani-Benito O. Ventura-León J. Caycho-Rodriguez T. (2019). Publicación científica de docentes que conforman el jurado dictaminador de tesis en una Facultad de Ciencias de la Salud peruana. *Rev. Cubana Inform. Ciencias Salud* 30:e1373.

[B29] Mamani-Benito O. Ventura-León J. Carranza R. Tito-Betancur M. Hilasaca-Mamani K. Rojas E. (2021a). Evidencias psicométricas iniciales de una Escala de Maltrato al Asesorado de Tesis (EMAT). *Educ. Medica* 22 298–304. 10.1016/j.edumed.2021.05.008

[B30] Mamani-Benito O. Verastegui-Diaz A. Mejia C. Caycho-Rodriguez T. (2020). Publicación científica de asesores de tesis de psicología de 30 universidades peruanas. *Rev. Int. Psicol.* 54:e1124. 10.30849/ripijp.v54i1.1124

[B31] Martin N. Cueli M. Cañamero L. Gonzales-Castro P. (2022). >Qué Sabemos Sobre los Estilos Educativos Parentales y los Trastornos en la Infancia y Adolescencia? Una Revisión de la Literatura. *Rev. Psicol. Educ.* 17 44–53. 10.23923/rpye2022.01.215

[B32] Mavrogalou-Foti A. P. Kambouri M. A. Çili S. (2024). The supervisory relationship as a predictor of mental health outcomes in doctoral students in the United Kingdom. *Front. Psychol*. 15:1437819. 10.3389/fpsyg.2024.1437819 39444829 PMC11497167

[B33] Mejia C. R. Mamani-Benito O. J. Condori S. Tito-Betancur M. Ramos G. Torres R. (2022). Producción Científica de los Asesores de Tesis de las Facultades de Medicina Humana en el Perú. *Gaceta Médica Boliviana* 45 45–50. 10.47993/gmb.v45i1.338

[B34] Miyahira J. M. (2023). >La tesis se considera publicación previa? *Rev. Méd. Herediana* 34 61–62. 10.20453/rmh.v34i3.4540

[B35] Muthén L. K. Muthén B. O. (2017). *Mplus User’s Guide*, 8th Edn. Los Angeles, CA: Muthén & Muthén.

[B36] Ooi P. B. Khor K. S. Tan C. C. Ong D. L. T. (2022). Depression, anxiety, stress, and satisfaction with life: Moderating role of interpersonal needs among university students. *Front. Public Health* 10:958884. 10.3389/fpubh.2022.958884 36249213 PMC9554619

[B37] Organizacion Mundial de la Salud. (2024). *La salud Mental de los Adolescentes.* Available online at: https://www.who.int/es/news-room/fact-sheets/detail/adolescent-mental-health (accessed December 12, 2025).

[B38] Peña-Tomas B. Serpa A. Caycho-Caja A. Escudero-Nolasco J. (2025). Estructura factorial, invarianza, sensibilidad y especificidad de la DASS-13 en población peruana. *Ciencias Psicol.* 19:e4257. 10.22235/cp.v19i1.4257

[B39] Peng S. Huang Y. (2024). Teachers’ authoritarian leadership and students’ well-being: the role of emotional exhaustion and narcissism. *BMC Psychol*. 12:590. 10.1186/s40359-024-02110-z 39449088 PMC11520137

[B40] Roman F. Santibañez P. Vinet E. (2016). Uso de las Escalas de Depresión Ansiedad Estrés (DASS-21) como Instrumento de Tamizaje en Jóvenes con Problemas Clínicos. *Acta Invest. Psicol.* 6 2325–2336.

[B41] Sultson H. Murd C. Havik M. Konstabel K. (2024). Negative affect instability predicts elevated depressive and generalized anxiety disorder symptoms even when negative affect intensity is controlled for: an ecological momentary assessment study. *Front. Psychol*. 15:1371115. 10.3389/fpsyg.2024.1371115 38716268 PMC11074391

[B42] Syahid A. Winna W. Kharima I. Sulliya L. Suryani Y. Komariah R. et al. (2023). The Correlation Of Excessive Assignment Between Student’s Mental Health. *JIPNAS* 1 147–151. 10.59435/jipnas.v1i3.197

[B43] Tapullima-Mori C. Chavez B. (2022). Escala de estrés, ansiedad y depresión (DASS-21): propiedades psicométricas en adultos de la provincia de San Martín. *Psiquemag* 11 73–88.

[B44] Tepper B. J. (2000). Consequences of abusive supervision. *Acad. Managem. J.* 43 178–190. 10.2307/1556375 23318324

[B45] Tolin D. F. O’Bryan E. M. Davies C. D. Diefenbach G. J. Johannesen J. (2023). Central and peripheral nervous system responses to chronic and paced hyperventilation in anxious and healthy subjects. *Biol. Psychol*. 176:108472. 10.1016/j.biopsycho.2022.108472 36481266 PMC9839632

[B46] Ventura-León J. Peña-Calero B. N. Burga-León A. (2023). The effect of normality and outliers on bivariate correlation coefficients in psychology: a Monte Carlo simulation. *J. Gen. Psychol*. 150 405–422. 10.1080/00221309.2022.2094310 35792742

[B47] Vilchez-Cornejo J. Viera-Morón R. D. Larico-Calla G. Alvarez-Cutipa D. C. Sánchez-Vicente J. C. Taminche-Canayo R. et al. (2020). Depression and abuse during medical internships in Peruvian hospitals. *Rev. Colomb. Psiquiatr.* 49 76–83. 10.1016/j.rcp.2018.08.001 32446423

[B48] Wilcox R. (2011). *Modern Statistics for the Social and Behavioral Sciences: A Practical Introduction*, 2nd Edn. Boca Raton, FL: CRC Press.

[B49] Yao Y. Chen J. Chi H. Hang Y. Qiao Z. (2025). Perceived abusive supervision and mental health among Chinese graduate students: the chain mediating roles of autonomy need and professional identity. *BMC Psychol*. 13:959. 10.1186/s40359-025-03324-5 40849637 PMC12374291

